# Workplace bullying and depressive symptoms among employees in Germany: prospective associations regarding severity and the role of the perpetrator

**DOI:** 10.1007/s00420-019-01492-7

**Published:** 2019-11-28

**Authors:** Stefanie Lange, Hermann Burr, Uwe Rose, Paul Maurice Conway

**Affiliations:** 1 Federal Institute for Occupational Safety and Health (BAuA), Nöldnerstraße 40-42, 10317 Berlin, Germany; 2 University of Copenhagen, Øster Farimagsgade 2A, 1353 Copenhagen K, Denmark

**Keywords:** Harassment, S-MGA, Mental health, Longitudinal design, Depression

## Abstract

**Objectives:**

The aim of this study was to investigate the effect of self-reported workplace bullying on depressive symptoms in a prospective study among a representative sample of employees from Germany. We focused specifically on the role of the perpetrator (co-workers and superiors), which was never done before in a longitudinal design.

**Methods:**

We used data from a nation-wide representative panel study with a 5-year follow-up (*N* = 2172). Data on bullying exposure were obtained separately for different perpetrators (co-workers and superiors) and degree of severity (severe bullying, i.e., at least weekly). Depressive symptoms were assessed with the Patient Health Questionnaire (PHQ). We used logistic regression analyses to examine the effect of workplace bullying at baseline on depressive symptoms at follow-up.

**Results:**

After adjusting for baseline depressive symptoms, severe bullying by co-workers significantly increased the 5-year risk of depressive symptoms (OR = 2.50). Severe bullying by superiors had a nonsignificant effect.

**Conclusions:**

Workplace bullying is a risk factor for depressive symptoms among employees in Germany. The type of perpetrator seems to be an important factor to consider, as indicated by the elevated risk of depressive symptoms when bullying is perpetrated by co-workers.

## Introduction

Bullying is a serious psychosocial risk factor in the workplace (Einarsen et al. 2011; Hauge et al. 2007). In Germany for example, the prevalence of severe bullying has been found to be 7% and of overall bullying 17% (Lange et al. 2019). Prevalence seems to be lower in, e.g., Scandinavian countries and higher in e.g. Great Britain and Turkey (Einarsen 2000). The negative effects of workplace bullying are far-reaching, with consequences for both individual health and the company’s performance (Bonde et al. 2016; Bowling and Beehr 2006; Conway et al. 2018; Einarsen and Nielsen 2014, 2015; Nielsen and Einarsen 2012). For example, bullying increases the risk for disability pensioning, poor mental health and burnout (Clausen et al. 2019; Conway et al. 2018). Although no common definition and operationalization of workplace bullying exist to date (Kemp 2014), it is agreed that the phenomenon takes place if an employee is persistently and repeatedly exposed to inappropriate treatment by one or more persons (Einarsen et al. 2011), and he/she finds it difficult to defend him/herself against the negative behavior (Conway et al. 2018; Hershcovis and Barling 2007). Thus, bullying might be equated with prolonged exposure to situations of emotional and social stress, a feeling of loss of control and inability to cope, which increases the risk of developing mental health problems (Reknes et al. 2016; Verkuil et al. 2010).

So far, only few prospective studies, mainly conducted in Scandinavia, have investigated the association between workplace bullying and depressive symptoms (e.g., Bonde et al. 2016; Einarsen and Nielsen 2015; Figueiredo-Ferraz et al. 2015; Gullander et al. 2014; Kivimaki et al. 2003; Rugulies et al. 2012). These studies showed a dose–response relationship, with frequent bullying yielding a higher risk for the development of depressive symptoms than occasional bullying.

An important, but rarely investigated question is whether this effect depends on the type of perpetrator (co-workers or superiors). An imbalance of power between targets and perpetrators is a central feature of bullying (Einarsen et al. 2003). While superiors have more formal power, co-workers might have more social power, i.e., they can influence social relationships and provoke social exclusion (Hershcovis and Barling 2010). Hershcovis and Barling (2010) found in their review that the magnitude of effects on attitudes, behaviors and health-related outcomes seems to differ strongly depending on perpetrator type. A possible explanation might be that bullying by different perpetrators could result in different response strategies from the affected employee and his or her organization. For example, bullying by superiors might result in the employees’ experience of job insecurity, while responses to bullying by co-workers may be more confrontational with less involvement of the company (Hershcovis and Barling 2010). Although previous studies examining the role of the perpetrator provided inconsistent results based on cross-sectional data (Hershcovis and Barling 2010; Török et al. 2016), we expect that formal power is more likely to create a power imbalance between perpetrator and affected employee and, thus, bullying by superiors might be more detrimental than bullying by co-workers. To test this hypothesis and to contribute bridging the mentioned research gaps, the present 5-year follow-up study sets out to investigate prospectively the effect of self-reported workplace bullying on depressive symptoms in a representative sample of employees in Germany, while also distinguishing by type of perpetrator.

## Methods

### Population

**Fig. 1:**
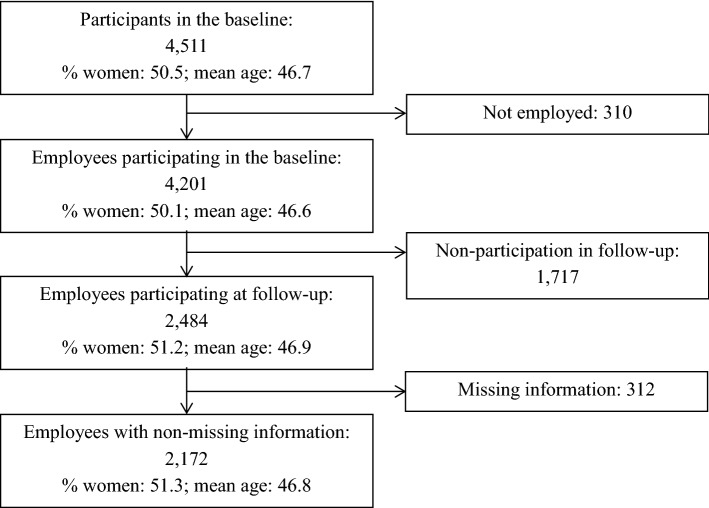
Flow diagram of participation

We used the Study on Mental Health at Work (S-MGA), a German nation-wide representative cohort (baseline 2011/2012, follow-up 2017). At baseline, the target population consisted of all employees in Germany born in 1951–1980 as of 31 December 2010 but excluding civil servants, self-employed individuals and freelancers (Rose et al. 2017). This multipurpose cohort was confined to these birth years because most people in employment are between 31 and 60 years, so they finished their education and training and are not yet retired. The design and sampling procedure of S-MGA are described in Rose et al. (2017). Of the 13,590 sampled addresses, 4511 participants completed the computer-assisted personal interview at baseline (response rate 33%). Of these, 4201 were employed at baseline (Fig. 1), of which 2484 participated at follow-up (follow-up rate 59%).

Younger age and low occupational status were related to more non-response at follow-up. Both the exposure variables (bullying by any perpetrator) and the outcome (baseline depressive symptoms) were unrelated to loss at follow-up (Table 1). We included only participants who were employed at baseline and did not have missing values, at both baseline and follow-up, for age, gender, socio-economic status, bullying and depressive symptoms (*N* = 2172).

**Table 1:** Baseline characteristics and comparison of respondents at follow-up, dropouts and analysis sample (without missing values)

	Employees at baseline		Dropouts due to non-participation		Analyzed sample	
	*N*	%	*N*	*%*	*N*	%
Gender						
Men	2096	50	885	52	1057	49
Women	2105	50	832	49	1115	51
Age						
31–40 years	1013	24	452	26	491	23
41–55 years	2543	61	997	58	1357	63
56–60 years	645	15	268	16	324	15
Occupation						
Unskilled workers	282	7	138	8	118	5
Skilled workers	1892	45	851	50	897	41
Semi-professionals	1099	26	413	24	616	28
Academics/managers	928	22	315	18	541	25
Bullying by co-workers						
No	3882	93	1582	93	2026	93
Occasional	177	4	78	5	83	4
Severe	119	3	48	3	63	3
Bullying by superiors						
No	3636	87	1473	86	1894	87
Occasional	346	8	151	9	177	8
Severe	200	5	82	5	101	5
Depressive symptoms						
No (PHQ < 10)	3457	92	1304	92	2010	93
Yes (PHQ ≥ 10)	288	8	111	8	162	8
Total	4201		1717		2172	

If percentages do not add to 100, this is due to rounding

### Measures

All information about the study variables was obtained through personal interviews in the respondents’ home (Rose et al. 2017). The only exception was depressive symptoms, which were measured through paper questionnaires that were filled in without the interviewers being present.

**Fig. 2:**
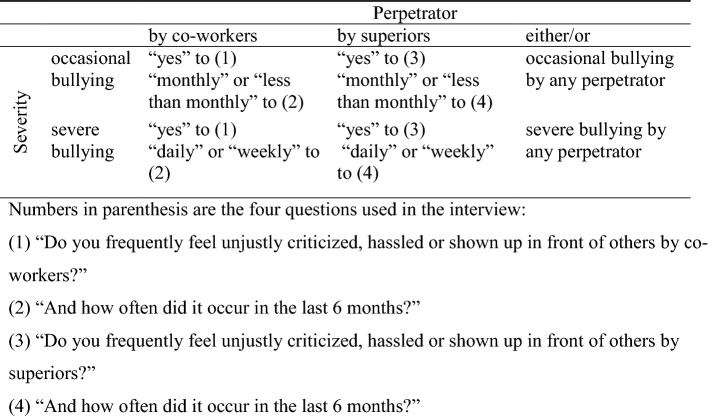
Classification based on perpetrator and severity

Workplace bullying was assessed using a hybrid approach, combining the behavioral experience and the self-labelling methods (Lange et al. 2018). Garthus-Niegel et al. (2016) showed that this scaling method had the same predictive validity as the reporting of negative acts based on the behavioral experience method. Participants were given two questions: (1) “Do you frequently feel unjustly criticized, hassled or shown up in front of others by co-workers?” and (3) “Do you frequently feel unjustly criticized, hassled or shown up in front of others by superiors?”, with the response options “yes” and “no”. Each question was followed by the question: (2, 4) “And how often did it occur in the last 6 months?” with the following response options: “daily”, “at least once a week”, “at least once a month” and “less than once a month”. By combining type of perpetrator and severity based on the cut-off proposed by Leymann (1996) (severe bullying: exposure to bullying once a week for at least 6 months), we formed the following six groups: severe bullying by co-workers and by superiors, occasional bullying by co-workers and by superiors, and severe and occasional bullying by any perpetrator (Fig. 2).

Depressive symptoms were assessed with the Patient Health Questionnaire with the questions (PHQ-9; Löwe et al. 2002): ‘Over the last 2 weeks, how often have you been bothered by any of the following problems?’’ The nine items in the scale were: ‘- Little interest or pleasure in doing things’, ‘- Feeling down, depressed or hopeless’ ‘Difficulty falling asleep or sleeping or increased sleep’, ‘Tiredness or feeling unable to have energy’, ‘Decreased appetite or excessive need to eat’, ‘Bad opinion of yourself’, ‘Difficulty concentrating on something’, ‘Slowed speech/movement or restlessness (“fidgety”)’, ‘Thoughts that you would rather be dead or want to self inflict pain’ with the response options ‘Not at all’ (0), ‘Several days’ (1), ‘More than half the days’ (2) and ‘Nearly every day’ (3). The scale score for depressive symptoms was determined as the sum of all available items (Spitzer et al. 1999). Cronbach’s Alpha was 0.81 at baseline, with inter-item correlations ranging from 0.21 to 0.51. We used the cut-off based on summed items scores, using a score of 10 or above as screening threshold for major depressive disorder (Manea et al. 2015).

Self-reported diagnosed depression was assessed through the following question only at follow-up: ‘Have you been diagnosed having a depression by a physician, a psychologist, or a psychotherapist since the last interview?’ (yes/no).

*Gender, age and socio-economic status*: We used baseline demographic information on gender, age and socio-economic status. The latter was measured using the International Standard Classification of Occupations (ISCO 08), with occupations categorized into four groups based on skill levels: unskilled workers, skilled workers, semi-professionals and academics/managers (TELEMATE 1999).

### Statistical analyses

We used logistic regression analyses to examine the association between workplace bullying at baseline and depressive symptoms at follow-up. We first calculated crude OR (Model 0). In Model 1, the ORs were adjusted for gender, age, and socio-economic status. We chose these covariates as potential confounders, as they have been shown to be associated with depressive symptoms (Thielen and Kroll 2013), and as two of them (age and socio-economic status) were associated with depressive symptoms (Lange et al. 2019). In Model 2, we additionally adjusted for depressive symptoms at baseline. This adjustment was done for three reasons: First, depressive symptoms at baseline are associated with depressive symptoms at follow-up; second, people with depressive symptoms might easier be victims of bullying (reverse causation) (Conway et al. 2018); and third, people with depressive symptoms might overreport bullying (Kolstad et al. 2011; Wang and Patten 2011; Zapf et al. 1996). Age was categorized as follows: 31–40, 41–55 and 56–60 years, to take into account a peak of the prevalence of depressive symptoms among German employees in the beginning of their 50’s (Thielen and Kroll 2013).

As studies relying on self-reports could inflate the association between working conditions and depression (Kolstad et al. 2011), we performed two sensitivity analyses. First, we used self-reported physician (or psychologist or psychotherapist)-diagnosed depression within the last 5 years as alternative outcome (*N* = 2168). Second, we repeated the main analyses for employees without depressive symptoms at baseline (*N* = 2010), because depressed employees at baseline could have evaluated their working conditions worse than their non-depressed counterparts (Zapf et al. 1996).

A third sensitivity analysis was performed to assess if prolonged exposure to workplace bullying had stronger effects on depressive symptoms. In this analysis, we repeated the main analyses after excluding all employees who changed their jobs within the study (*N* = 1627). The statistical analyses were conducted using IBM SPSS Statistics 24.

## Results

### Logistic regression analyses

The prospective association between bullying (stratified by perpetrator and severity) and depressive symptoms is shown in Table 2. Of the 63 employees being severely bullied by their co-workers at baseline, 16 (25%) reported depressive symptoms at follow-up. Regarding severe bullying by superiors, 24 out of 101 (24%) reported depressive symptoms at follow-up. The logistic regression, adjusted for sociodemographic characteristics (Model 1), showed consistently higher ORs for depressive symptoms for each combination of perpetrator and severity when contrasted to not being bullied, except for occasional bullying by superiors. After additionally adjusting for baseline depressive symptoms, all OR’s decreased in size and only severe bullying by co-workers was still significantly associated with depressive symptoms (OR = 2.50; CI 95% = 1.29–4.85). When not distinguishing by perpetrator (either/or), the same dose–response association could be observed regarding bullying by co-workers: specifically, the association was significant for severe bullying (OR = 1.71; CI 95% = 1.04–2.82). However, note that the confidence intervals between occasional and severe bullying overlap.

**Table 2:** Prospective models for exposure to workplace bullying at baseline (2011/12) and depressive symptoms at follow-up (2017) by type of perpetrator and severity

	*N*	Cases	Cases (%)	Self-reported depressive symptoms (PHQ sum ≥ 10)					
				Model 0		Model 1		Model 2	
				*p* value*	OR (95% CI)	*p* value*	OR (95% CI)	*p* value*	OR (95% CI)
Bullying by co-workers				< 0.001		< 0.001		0.017	
No	2026	186	9		1		1		1
Occasional	83	14	17		2.01 (1.11–3.64)		1.82 (1.00–3.32)		1.45 (0.75–2.84)
Severe	63	16	25		3.37 (1.87–6.06)		3.33 (1.83–6.05)		2.50 (1.29–4.85)
Bullying by superiors				< 0.001		< 0.001		0.315	
No	1894	170	9		1		1		1
Occasional	177	22	12		1.44 (0.90–2.31)		1.45 (0.90–2.34)		1.10 (0.65–1.85)
Severe	101	24	24		3.16 (1.95–5.13)		3.26 (1.99–5.35)		1.55 (0.88–2.74)
Bullying by either/or				< 0.001		< 0.001		0.085	
No	1816	156	9		1		1		1
Occasional	216	29	13		1.65 (1.08–2.52)		1.62 (1.05–2.50)		1.27 (0.80–2.02)
Severe	140	31	22		3.03 (1.97–4.66)		3.06 (1.97–4.75)		1.71 (1.04–2.82)

*N* = 2172

Model 0: Unadjusted model. Each bullying variable was introduced separately in the model

Model 1: Adjusted for gender, age and socio-economic status. Each bullying variable was introduced separately in the model

Model 2: Adjusted for gender, age, socio-economic status and PHQ at baseline. Each bullying variable was introduced separately in the model

*This *p* value denotes to what extent the whole categorical bullying variable is associated with self-reported depressive symptoms

We also looked at employees being severely bullied by both co-workers and superiors (Table not shown). Of the 24 employees with double exposure, 9 (38%) reported depressive symptoms at follow-up in the fully adjusted model (OR = 3.38; CI 95% = 1.27–9.00), but one has to be aware of the limited number of observations.

### Sensitivity analyses

In the first sensitivity analysis, we repeated the logistic regression analysis on the same sample but using diagnosed depression as outcome (*N* = 2168). After adjusting for gender, age, socio-economic status and depressive symptoms at baseline, only severe bullying by co-workers showed a significantly elevated risk for diagnosed depression (OR = 2.06, CI 95% = 1.01–4.18; Table 3 in Appendix A).

In the second sensitivity analysis, we repeated the main analyses for employees without depressive symptoms at baseline (*N* = 2010). The dose–response relationship, as well as the stronger effect of co-workers, was still observable, although we could not obtain statistical significance. This may be due the limited statistical power (Table 4 in Appendix B) as the second sensitivity analysis relied on a smaller sample. When disregarding perpetrator type, however, the power was sufficient and significant OR’s were obtained for both occasional and severe bullying.

The third sensitivity analysis was based on employees who did not change their job between 2011/12 and 2017 (*N* = 1627) to see if prolonged exposure to bullying had stronger effects. In the fully adjusted model, the ORs were higher than in the main analyses for both types of perpetrators; even bullying by superiors showed significant effects on depressive symptoms (bullying by co-workers: OR = 2.93, CI 95% = 1.37–6.29; bullying by superiors: OR = 2.33, CI 95% = 1.18–4.60; Table 5 in Appendix C). Note that this analysis might oversee people who left work due to depressive symptoms caused by previous bullying.

## Discussion

The present study, conducted on a representative sample of German employees, did not confirm that bullying by superiors was more detrimental for the development of depressive symptoms than bullying by colleagues. Bullying by colleagues had in fact a higher risk than bullying by superiors, but this difference was not significant. To our knowledge, this is the first study examining the role of the perpetrator in a longitudinal design. In addition, we could corroborate the results of six—mainly Scandinavian—longitudinal studies focusing on depression or depressive symptoms (Theorell et al. 2015) which found that workplace bullying (regardless of perpetrator type) is a risk factor for depressive symptoms (Bonde et al. 2016; Einarsen and Nielsen 2015; Figueiredo-Ferraz et al. 2015; Gullander et al. 2014; Kivimaki et al. 2003; Rugulies et al. 2012). A dose–response relationship was also confirmed (Bonde et al. 2016; Figueiredo-Ferraz et al. 2015; Gullander et al. 2014; Rugulies et al. 2012).

Neither occasional bullying nor severe bullying by superiors showed a significant effect after 5 years. However, the risk for depressive symptoms at follow-up was two-and-a-half times higher among employees being severely bullied by co-workers than among their non-bullied counterparts. This effect is remarkable if one considers the long follow-up period. Our estimates of the association between bullying and depressive symptoms may be conservative, as some employees who have experienced depressive symptoms because of bullying may have recovered before follow-up. After excluding participants with depressive symptoms at baseline, we still found indications of dose–response relationships and the role of co-workers as most impactful perpetrators, although the associations were not significant due to reduced statistical power. However, the main analysis did have sufficient power; so, the absence of effects regarding severe bullying by superiors cannot be explained by power issues.

The stronger effect of severe bullying by co-workers is only visible after adjusting for baseline depressive symptoms and in the second sensitivity analysis without participants with baseline depressive symptoms. There are at least two different interpretations. It might be that coping strategies of depressed employees are sufficient for handling bullying by superiors but not for dealing with bullying by co-workers. If this would be true, the more detrimental effect of bullying by co-workers would be exclusively linked to the affected employee’s health. It might also be that baseline depressive symptoms are caused by bullying before baseline; so, the stronger effect of bullying by co-workers would be a consequence of prolonged exposure. To further examine these mechanisms and to get to know how long employees experienced bullying before baseline, studies with three or more waves are needed.

In the analysis in which we aimed to look at prolonged exposure to bullying and thus confined the analysis only to those employees who worked in the same job at both baseline and follow-up, the dose–response and the stronger effect of bullying by co-workers were confirmed. As expected, bullying had stronger effects in this smaller sample. Especially bullying by superiors had stronger and in this case significant effects. This may indicate that turnover partly prevents, or at least weakens, the detrimental effects of bullying by superiors, but not that by co-workers. It could also be that the effects of bullying by co-workers are more lasting and less reversible than those of bullying by superiors. Note, that by excluding employees who left their work, we have also probably excluded bullied employees who developed depressive symptoms and left their work due to previous bullying. Thus, the true risks could be even higher. Future studies are needed that look into the mechanisms behind these differential effects and as mentioned before more than two waves would be preferable (Beltagy et al. 2018).

### Role of the perpetrator

Török et al. (2016) observed a stronger association with depression for bullying by superiors in a cross-sectional study, while in their meta-analysis Hershcovis and Barling (2010) did not find any difference depending on perpetrator type. In contrast to these previous findings, our study showed that, in a representative sample of employees in Germany, bullying by superiors did not pose a higher risk for depressive symptoms than bullying by colleagues. This result was confirmed in two sensitivity analyses using physician-diagnosed depression and excluding participants with depressive symptoms at baseline. The discrepant results obtained in our study and in the study by Török et al. (2016) might be explained by the different distribution of perpetrators in Germany and in Scandinavia. In Scandinavia, the most frequent perpetrators are co-workers, while in other European countries, including Germany, bullying is more often enacted by superiors (Einarsen 2000; Lange et al. 2018; Ortega et al. 2009). People may endure effects that are more serious when the type of exposure is less common, due to missing habituation (Bondü et al. 2016; Vickers 2010). In line with this, it might be that bullying by colleagues has a stronger negative effect in Germany because employees do not expect being bullied by this group; while in Scandinavia, the same applies to bullying by superiors. So, power imbalance might not be sufficient to understand this, habituation might also play a role. Indeed, compared to continental Europe, hierarchies are flatter in Scandinavia and power is less dependent on the formal position (Einarsen 2000). As indicated the point estimates of risk for bullying by co-workers in the present study was generally higher than the point estimates of risk for bullying by superiors but both were within the same confidence intervals. To examine if the risks for bullying by different perpetrators truly deviate from each other, higher powered studies are needed.

### Methodological considerations

A main strength of the present study is that it is the first in the German context examining the association between workplace bullying and depressive symptoms using a longitudinal design. This is also the first study investigating the role of the perpetrator in a longitudinal perspective. Additionally, the sample was representative for all employees born in 1951–1980 in Germany (except for civil servants, self-employed individuals and freelancers).

There are, however, some limitations worth mentioning. The study’s response rate is rather low; but the participating population was in general representative due to social background, region, age, gender and other demographic traits. It might, however, be that people with depressive symptoms take part in the study to a lesser extent, so that people developing depressive symptoms as a result of bullying do not respond to the second interview in this study. Maybe this response bias would lead to more conservative results in the present study. In the S-MGA-cohort, people with self-rated health other than ‘very good’ at baseline had a 25% higher risk of non-participation at follow-up (Schiel et al. 2018). Another issue is that it might be that people with depressive symptoms are at higher risk for being bullied (Conway et al. 2018). Therefore, we in the main analysis controlled for depressive symptoms at baseline. There is a need for multiwave studies to assess this issue of reverse causation better. A depressive mood might bias self-reported measures (Kolstad et al. 2011; Wang and Patten 2011; Zapf et al. 1996). An advantage of our study is that bullying and depressive symptoms were not obtained with the same method. Specifically, information about bullying was collected through personal interviews, while depressive symptoms were assessed using a paper questionnaire, although the source of this information was the same (namely the participant). The examined association was confirmed, even if for bullying by co-workers only, also when using physician-diagnosed depression as alternative outcome. However, there might be a higher overlap between this alternative outcome and baseline bullying because the PHQ measures current symptoms (last 2 weeks) and the diagnosis of depression could have been anytime within the last 5 years between baseline and follow-up-measurements. Thus, it is also possible that the depression was diagnosed only few weeks after baseline and the association with baseline bullying would then overestimate the effect due to the cross-sectional nature of the analysis.

It could be seen as drawback that we measured severity by frequency but our results clearly showed higher effects on depressive symptoms if bullying occurs more frequent. Thus, frequency seems to be a useful proxy measure of severity and is also used by other authors (e.g., Gullander et al. 2014; Rugulies et al. 2012).

The sample did not include employees younger than 31 years of age and, due to limited statistical power, we could not examine if the association between bullying and depressive symptoms would be stronger in certain age groups. Additionally, our sample did not include occupational groups such as civil servants, self-employed individuals and freelancers.

Although we found an effect of severe bullying by co-workers on depressive symptoms, the long interval between baseline and follow-up may limit the detection of possible effects of severe bullying by superiors or occasional bullying by any perpetrator. As earlier indicated, it could be that some employees may have recovered before follow-up after experiencing depressive symptoms because of bullying. Future studies should examine the effect using shorter follow-up intervals.

It might also be that we overstate the effect of bullying when controlling for social class with only four categories (residual confounding). To overcome this, one would need a finer classification of occupations according to qualification levels than what is presently available (Hagen 2015; TELEMATE 1999). An alternative would be to use income as a proxy for social class, but this poses a range of methodological problems regarding, e.g., non-response and how to treat part time employed, which constitute a large part of especially female employees in Germany (Eurostat 2016). We cannot rule out further residual confounding caused by possible risk factors for depressive symptoms, such as, for instance, psychosocial working conditions other than workplace bullying. We decided not to include other psychosocial confounders both for power considerations and for the fact that psychosocial working conditions such as low social support, poor quality of leadership or high quantitative demands, are established risk factors for workplace bullying (Balducci et al. 2018), and controlling for them could have introduced overadjustment in our analyses.

### Comparison with other studies

Comparing our results with previous research is difficult because of a host of methodological differences. First, most other studies, except one (Einarsen and Nielsen 2014), used shorter follow-up intervals (e.g., 2 years). Moreover, only Gullander et al. (2014) and Rugulies et al. (2012) used comparable classifications of bullying frequency (occasional bullying: monthly or less; severe bullying: weekly or daily). In contrast to our results, both studies reported significant odds ratios for occasional bullying, which might point to rather short-term effects of occasional bullying in comparison to severe bullying. Perhaps, a depressive mood enhanced by occasional bullying can be overcome more quickly than in case of more serious forms of bullying. Sample differences could also explain the discrepant findings. For example, the study by Gullander et al. (2014) was composed mainly of female participants (75%) and Rugulies et al. (2012) included female eldercare workers only; so, differences in occupational distributions between genders may play a role (Boje and Furåker 2002). It is also noticeable that the odds ratios for severe bullying in the studies of Gullander (9.63 [CI 95% 3.42–27.10]) and Rugulies (8.45 [CI 95% 4.04–17.70]) were stronger than those obtained in our study (e.g., 2.43 [CI 95% 1.25–4.72] for bullying by co-workers) and showed large confidence intervals. This could be due to the shorter follow-up and to the rather low number of participants severely bullied at baseline and with depressive symptoms at follow-up in both studies (Gullander 6; Rugulies 9; our study: 16 for bullying by co-workers and 24 for bullying by superiors).

Further differences between studies that might be worth considering are mode of data collection and the origin of the sample: indeed, other studies were mainly conducted using postal questionnaires and in Scandinavian countries, while we collected data in Germany employing face-to-face interviews. Compared to postal questionnaires, responses to personal interviews could be biased by social desirability issues (Krumpal 2013). Finally, the welfare state regime in Germany might be able to buffer the effect of poor working conditions to a lower degree than in Scandinavian countries (Dragano et al. 2011).

## Conclusion and perspectives

We were the first to investigate prospectively the impact of self-reported workplace bullying on depressive symptoms in a sample of employees in Germany. Similarly, we were the first to distinguish by the type of perpetrator longitudinally. We found that weekly or daily bullying by co-workers and superiors both increased the risk of depressive symptoms after 5 years, whereas monthly or less than monthly bullying showed weaker associations.

Although bullying by superiors is more prevalent in the workplace and although superiors have more formal power than colleagues, our results did not find that bullying by superiors was more detrimental for employees’ mental health than bullying by colleagues—the opposite might even be the case. In more well-powered studies—preferably with more waves—the different effects of bullying by different perpetrators should be statistically tested and then, it would be of interest to delve into the mechanisms behind these differential effects.

As expected (Figueiredo-Ferraz et al. 2015; Gullander et al. 2014; Rugulies et al. 2012), we observed a strong dose–response gradient indicating “at least weekly” as a proper cut-off for identifying risk groups. Thus, it is recommendable to include an assessment of frequency in future analyses of bullying, while avoiding a simple dichotomization of the exposure.

Future studies should use a three-wave design to strengthen the possibility to draw causal conclusions about the studied relationship. An investigation including employees free from bullying and depressive symptoms in the first wave, becoming subjected to bullying in the second wave (still without depressive symptoms), and developing depressive symptoms in the third wave, would provide stronger etiologic evidence. Finally, effects of bullying should also be investigated among younger employees.

## Appendix A

See Table 3.

**Table 3:** Prospective models for exposure to workplace bullying at baseline (2011/12) and physician-diagnosed depression between baseline and follow-up (2017) by type of perpetrator and severity

	*N*	Cases	Cases (%)	Self-reported physician-diagnosed depression					
				Model 0		Model 1		Model 2	
				*p* value*	OR (95% CI)	*p* value*	OR (95% CI)	*p* value*	OR (95% CI)
Bullying by co-workers				0.001		0.002		< 0.001	
No	2024	166	8		1		1		1
Occasional	82	13	16		2.11 (1.14–3.90)		1.98 (1.06–3.67)		1.60 (0.82–3.13)
Severe	62	12	19		2.69 (1.40–5.14)		2.71 (1.40–5.24)		2.06 (1.01–4.18)
Bullying by superiors				< 0.001		< 0.001		0.255	
No	1893	150	8		1		1		1
Occasional	176	22	13		1.66 (1.03–2.67)		1.71 (1.05–2.76)		1.37 (0.82–2.28)
Severe	99	19	19		2.76 (1.63–4.68)		2.86 (1.67–4.89)		1.48 (0.81–2.68)
Bullying by either/or				0.016		< 0.001		0.155	
No	1815	140	8		1		1		1
Occasional	215	27	13		1.72 (1.11–2.66)		1.73 (1.11–2.70)		1.41 (0.88–2.25)
Severe	138	24	17		2.52 (1.57–4.04)		2.59 (1.60–4.18)		1.52 (0.89–2.58)

*N* = 2168

Model 0: Unadjusted model. Each bullying variable was introduced separately in the model

Model 1: Adjusted for gender, age and socio-economic status. Each bullying variable was introduced separately in the model

Model 2: Adjusted for gender, age, socio-economic status and PHQ at baseline. Each bullying variable was introduced separately in the model

*This *p* value denotes to what extent the whole categorical bullying variable is associated with physician-diagnosed depression

## Appendix B

See Table 4.

**Table 4:** Prospective models for exposure to workplace bullying at baseline (2011/12) and depressive symptoms at follow-up (2017) by type of perpetrator and severity. Participants with baseline depressive symptoms (PHQ sum score ≥ 10) were excluded

	*N*	Cases	Cases (%)	Self-reported depressive symptoms (PHQ sum >=10)			
				Model 0		Model 1	
				*p* value*	OR (95% CI)	*p* value*	OR (95% CI)
Bullying by co-workers				0.109		0.138	
No	1889	129	7		1		1
Occasional	71	7	10		1.49 (0.67–3.32)		1.40 (0.63–3.14)
Severe	50	7	14		2.22 (0.98–5.04)		2.18 (0.96–4.99)
Bullying by superiors				0.231		0.205	
No	1788	121	7		1		1
Occasional	154	15	10		1.49 (0.85–2.61)		1.52 (0.86–2.68)
Severe	68	7	10		1.58 (0.71–3.53)		1.61 (0.72–3.64)
Bullying by either/or				0.023		0.024	
No	1718	111	7		1		1
Occasional	189	20	11		1.71 (1.04–2.83)		1.71 (1.03–2.84)
Severe	103	12	12		1.91 (1.02–3.59)		1.93 (1.01–3.66)

*N* = 2010

Model 0: Unadjusted model. Each bullying variable was introduced separately in the model

Model 1: Adjusted for gender, age, and socio-economic status. Each bullying variable was introduced separately in the model

*This *p* value denotes to what extent the whole categorical bullying variable is associated with self-reported depressive symptoms

## Appendix C

See Table 5.

**Table 5:** Prospective models for exposure to workplace bullying at baseline (2011/12) and depressive symptoms at follow-up (2017) by type of perpetrator and severity. Participants who changed their jobs were excluded

	*N*	Cases	Cases (%)	Self-reported depressive symptoms (PHQ sum >=10)					
				Model 0		Model 1		Model 2	
				*p* value*	OR (95% CI)	*p* value*	OR (95% CI)	*p* value*	OR (95% CI)
Bullying by co-workers				< 0.001		< 0.001		0.009	
No	1519	139	9		1		1		1
Occasional	64	11	17		2.06 (1.05–4.04)		1.92 (0.97–3.80)		1.77 (0.85–3.70)
Severe	44	12	27		3.72 (1.88–7.39)		3.54 (1.77–7.11)		2.93 (1.37–6.29)
Bullying by superiors				< 0.001		< 0.001		0.048	
No	1450	132	9		1		1		1
Occasional	118	13	11		1.24 (0.68–2.26)		1.22 (0.66–2.24)		0.97 (0.50–1.87)
Severe	59	17	29		4.04 (2.24–7.30)		3.92 (2.15–7.15)		2.33 (1.18–4.60)
Bullying by either/or				< 0.001		< 0.001		0.025	
No	1389	121	9		1		1		1
Occasional	148	19	13		1.54 (0.92–2.59)		1.49 (0.88–2.51)		1.24 (0.71–2.17)
Severe	90	22	24		3.39 (2.02–5.68)		3.28 (1.94–5.54)		2.22 (1.24–3.98)

*N* = 1627

Model 0: Unadjusted model. Each bullying variable was introduced separately in the model

Model 1: Adjusted for gender, age and socio-economic status. Each bullying variable was introduced separately in the model

Model 2: Adjusted for gender, age, socio-economic status and PHQ at baseline. Each bullying variable was introduced separately in the model

*This *p* value denotes to what extent the whole categorical bullying variable is associated with self-reported depressive symptoms
